# Clinical trial to evaluate pharmacokinetics and pharmacodynamics of medroxyprogesterone acetate after subcutaneous administration of Depo-Provera

**DOI:** 10.1016/j.fertnstert.2020.11.002

**Published:** 2021-04

**Authors:** Vera Halpern, Vivian Brache, Douglas Taylor, Anja Lendvay, Leila Cochón, Jeffrey T. Jensen, Laneta J. Dorflinger

**Affiliations:** a FHI 360, Durham, North Carolina; b Profamilia, Biomedical Research Department, Santo Domingo, Dominican Republic; c Department of Obstetrics and Gynecology, Oregon Health and Science University, Portland, Oregon

**Keywords:** Depot medroxyprogesterone acetate, subcutaneous, contraception, pharmacokinetics, suppression of ovulation

## Abstract

**Objective:**

To evaluate the pharmacokinetics and pharmacodynamics of medroxyprogesterone acetate after a single subcutaneous injection in the abdomen of 150 or 300 mg Depo-Provera and compare results to two injections of Depo-SubQ Provera 104 given 3 months apart.

**Design:**

Partially randomized, multicenter, parallel-group study.

**Setting:**

Research unit.

**Patient(s):**

Forty-two women of reproductive age with confirmed ovulatory cycle and body mass index of 18–35 kg/m^2^.

**Intervention(s):**

Women received a single subcutaneous injection of 150 mg (n = 24) or 300 mg (n = 9) of Depo-Provera or two injections of Depo-SubQ Provera 104 (n = 9).

**Main Outcome Measure(s):**

Suppression of ovulation as measured by progesterone, serum medroxyprogesterone acetate concentrations, and estimated pharmacokinetics parameters.

**Result(s):**

No ovulations were observed during 7 months after a single injection of 150 or 300 mg Depo-Provera. The 150 mg group had a similar C_max_ as observed over two injection cycles of Depo-SubQ Provera 104 and a similar 6-month trough concentration as the 3-month trough of Depo-SubQ Provera 104.

**Conclusion(s):**

Our pharmacodynamics and pharmacokinetics data provide proof of concept that Depo-Provera (150 mg) may be an effective contraceptive method when injected subcutaneously every 6 months, with up to a 4-week grace period for reinjections.

**Clinical Trial Registration Number:**

NCT02456584.

## Abstract (es)

Ensayo clínico para la evaluación de la farmacocinética y farmacodinámica del acetato de medroxiprogesterona tras la administración subcutánea de Depo-Provera.

**Objetivo:**

Evaluar la farmacocinética y farmacodinámica de acetato de medroxiprogesterona después de una única inyección subcutánea abdominal de 150 o 300 mg de Depo-Provera y comparar los resultados con dos inyecciones de Depo-SubQ Provera 104 administradas con un intervalo de 3 meses.

**Diseño:**

Estudio multicéntrico, parcialmente aleatorizado y en grupos paralelos.

**Entorno:**

Unidad de investigación.

**Paciente(s):**

Cuarenta y dos mujeres en edad reproductiva con ciclos ovulatorios confirmados e índice de masa corporal entre 18 y 35 kg/m2.

**Intervención:**

Las mujeres recibieron una única inyección subcutánea de 150 mg (n=24) o 300 mg (n=9) de Depo-Provera o dos inyecciones de Depo-SubQ Provera 104 (n=9).

**Medidas principales de resultado(s):**

Supresión de la ovulación determinada por progesterona, concentraciones séricas de acetato de medroxyprogesterona y estimación de los parámetros farmacocinéticos.

**Resultado(s):**

No se observaron ovulaciones durante 7 meses después de una única inyección de 150 o 300 mg de Depo-Provera. El grupo de 150 mg tuvo una Cmax similar a la observada con dos ciclos de inyección de Depo-SubQ Provera 104 y una concentración similar durante 6 meses a la de Depo.SubQ Provera 104 durante 3 meses.

**Conclusión(es):**

Nuestros datos farmacodinámicos y farmacocinéticos proporcionan la “prueba de concepto” de que la Depo-Provera (150 mg) puede ser un método contraceptivo eficaz inyectado cada 6 meses vía subcutánea, con hasta 4 semanas de período de gracia para las re-inyecciones.

**Palabra(s) clave(s):**

Medroxiprogesterona acetato depot, subcutánea, contracepción, farmacocinética, supresión de la ovulación.

**Discuss:** You can discuss this article with its authors and other readers at **https://www.fertstertdialog.com/posts/31238**

Injectable contraception has been a cornerstone of international family planning programs for decades. An estimated 74 million women worldwide use injectable contraceptives to prevent pregnancy (1). Depending on the formulation, currently available injectables are effective for 1–3 months, requiring women to return to their provider monthly or quarterly. A need for frequent reinjections is recognized as a significant disadvantage of this method (2). Previous research has demonstrated that injectable contraceptives with a longer interval between injections led to better adherence and continuation rates when compared with those with shorter intervals (3, 4). A longer-acting injectable could facilitate use, improve continuation, possibly increase effectiveness, and provide women with greater choice. Six months is a target duration from a safety perspective for a hormonal method that cannot be discontinued immediately in case of unwanted side effects or emerging medical contraindications and is preferred by both users and providers (5). While efforts are under way to develop novel sustained drug delivery technologies that hold promise for the development of a new 6-month injectable contraceptive product (6, 7), the adaptation of existing methods for novel uses is an appealing, cost-efficient approach to expedite availability of new contraceptive options.

The subcutaneous (SC) route of administration, characterized by slower absorption and a more sustained release when compared with intramuscular injection, provides a path toward lowering the effective dose of a drug (8). By changing the route of administration, Pfizer developed Depo-SubQ Provera 104 (104 mg/0.65 mL) for SC injection (Depo-SubQ 104) that is similar to the 3-month Depo-Provera (150 mg/mL) for intramuscular use (Depo-Provera) but uses 30% less drug (9, 10). The same conceptual approach may be applied to extend the duration of action without increasing the dose. In the Pfizer dose-finding study, the average medroxyprogesterone acetate (MPA) concentrations at day 112 after 100 and 150 mg doses were well above 0.2 ng/mL (the threshold presumed necessary to exert a consistent contraceptive effect) (8, 11). According to the labeling of Depo-SubQ 104, mean serum concentrations of MPA remain within the contraceptive range 150 days after a single injection of that product. Based on these findings, we hypothesized that a 44% increase of the SC dose (i.e., from 104 to 150 mg) may result in concentrations of MPA that are consistent with suppression of ovulation for at least 6 months and that 150 mg of Depo-Provera has a potential to provide 6 months of contraceptive protection if injected SC. We conducted a pharmacokinetic (PK) and pharmacodynamic (PD) study of 150 mg and 300 mg of Depo-Provera injected via off-label SC route, to identify the lowest of the two doses that effectively suppresses ovulation for at least 6 months and has a PK profile consistent with contraceptive effect for 6 months.

## Materials and methods

This partially randomized, parallel-group study (clinicaltrials.gov no: NCT02456584) was conducted at Biomedical Research Department at PROFAMILIA (Santo Domingo, Dominican Republic [DR]) and the Women’s Health Research Unit at Oregon Health and Science University (OHSU; Portland, OR) between September 2015 and May 2018. The study was approved by the Comite de Etica Profamilia, CONABIOS, the FHI 360 Protection of Human Subjects Committee, and the OHSU institutional review board. All women provided informed consent before entering the study.

To be enrolled in the study, women had to be 18–40 years old, not pregnant, and at low risk of pregnancy (sterilized, in exclusively same-gender partnership, in monogamous relationship with vasectomized partner, abstinent, using nonhormonal intrauterine device, or consistently using condoms) and have a body mass index (BMI) of 18–35 kg/m^2^. Ovulation was confirmed before study enrollment in all women by two consecutive progesterone (P) measurements ≥ 4.7 ng/mL obtained within 5 days. We excluded women who had medical contraindications (12); were allergic to MPA; were recently pregnant (within 3 months); used oral contraceptive pills, implants, or hormonal intrauterine system in the month before enrollment; or used MPA injectables in the last 12 months or a combined injectable in the last 6 months.

We planned to randomize 48 eligible participants in a 2:1:1 allocation ratio to receive a single injection of 150 mg (1 mL) Depo-Provera (150 mg group), a single injection of 300 mg (2 mL) Depo-Provera (300 mg group), or two injections of Depo-SubQ 104 (reference group) at enrollment and 3 months later. The randomization sequence (stratified on investigational site and using a block size of 4) was generated using SAS/STAT software, and treatment allocation was concealed using sequentially numbered opaque randomization envelopes.

The study was not blinded due to differences in volume and packaging of the study drugs as well as differences in the schedule of injections and follow-up visits between the treatment groups after day 7 postinjection. However, the laboratory personnel conducting MPA and P testing remained blinded to treatment assignments throughout the study.

Treatment was initiated in all three groups within the first 5 days of the menstrual cycle. All injections were administered SC in the abdomen after the SC injection technique recommended by Pfizer (9). To maintain the same depth of injection in all study groups, Depo-Provera was administered through a 26-gauge 3/8-inch needle, the needle size provided with the prefilled Depo-SubQ 104 syringe. We measured blood pressure, height, and weight and collected blood samples for MPA, P, estradiol (E_2_), and cortisol before injection.

The primary PD endpoint was time to ovulation where ovulation was defined as a single elevated serum P ≥ 4.7 ng/mL (participants with elevated P were asked but not required to return within 5 days for a confirmatory measurement). Participants were monitored until they ovulated or for 12 months, whichever was earlier, but for a minimum of 7.5 months after treatment initiation. Serum P and E_2_ were measured in the 150 and 300 mg test groups on days 0 (baseline), 7, and 14; then every 2 weeks until week 12; then approximately weekly until return of ovulation, or through month 12, whichever was earlier. In the reference group serum P and E_2_ were measured following the same schedule as in the 150 and 300 mg through week 12. After the second injection at week 13, P and E_2_ were measured every two weeks through week 25, and then approximately weekly until return of ovulation, or through month 12, whichever was earlier.

We expected that women in the 150 mg/mL group would have the greatest chance of an ovulatory response in the treatment period. For this reason, we also measured follicle growth and outcome by transvaginal sonogram (TVS) in the 150 mg group twice a week between weeks 22 and 32 (Supplemental Fig. 1). For the purpose of this protocol a lead (or dominant) follicle was defined as a follicle with the mean diameter of 12 mm or more. Follicle rupture was defined as abrupt disappearance or a reduction in size of at least 50% of the echo image of the lead follicle. The supportive PD parameters, including confirmation of elevated P, E_2_, and TVS results, were not used to modify the primary definition of ovulation but rather to more accurately characterize ovarian function.

The secondary PK outcome was estimated based on serum MPA levels that were measured in the test groups on days 0, 1, 2, 3, 5, 7, 10, 12, 14, 18; then at weeks 3, 4, 5, 6, 8, 10, 12, 13, 15, 17, 19, 21, 23, 25, 26, 28, 30, 32; then every 28 days until return of ovulation, or month 12, whichever was earlier. In the reference group the sampling schedule differed slightly to measure MPA levels around the time of the second injection. If ovulation had not returned by month 12, MPA was measured at weeks 61 and again at week 74 if ovulation was not detected earlier.

Safety was assessed throughout the study by monitoring vital signs (blood pressure and pulse), weight, use of concomitant medications, occurrence of adverse events, and injections site reactions. Bleeding was assessed at day 7, months 3 and 7.5, and at the final visit through questions about changes in bleeding pattern since last visit (e.g., spotting, regular, irregular, or no bleeding at all), and whether the participant found her new bleeding pattern acceptable. Possible suppression of adrenal function due to the MPA glucocorticoid-like effect was evaluated by measuring serum cortisol at baseline and treatment days 5, 7, 14, 42, and month 3 and 7.5. Acceptability was assessed at the end of the injection visit, at day 7, months 3 and 7.5, and at the final visit through questions about likes and dislikes of the method, preferred reinjection frequency, and whether participants would use this method for contraception if it was found to be effective for 6 months. Women with delayed return to ovulation (more than 12 months from treatment initiation) were monitored by measuring P and E_2_ weekly up to 5 times during month 15 and again during month 18 if ovulation was not detected earlier.

We considered the target sample size of 48 participants (12 women each in the reference and 300 mg groups and 24 in the 150 mg group) sufficient to provide meaningful insights into the distributions of PK and PD outcomes (8, 11, 13). The larger 150 mg group size allowed us to increase the precision with which to estimate the distribution of return to ovulation in the lowest dose group, which we hypothesized had the potential to provide 6 months of contraceptive protection. The PK and PD analyses were performed on the primary evaluable population that excluded participants with MPA at baseline exceeding 5% of their C_max_ or with major protocol violations that could interfere with interpretation of the PK and PD data.

We estimated the cumulative probability of return to ovulation based on the Kaplan-Meier product-limit method, with 95% confidence intervals derived using the complementary log-log transformation. The PK of MPA was evaluated based on individual serum MPA concentration-time profiles and estimated noncompartment PK parameters. For individuals in the test groups, C_max_, T_max_, C_182_, C_210_, AUC_(0-91)_, AUC_(0-182)_, AUC_(0-210)_, AUC_(0-∞)_, and the apparent terminal half-life were estimated. In the reference group the following PK parameters were estimated: C_max_ and T_max_ (by injection cycle), C_91_, C_182_, C_210_, AUC_(0-91)_, AUC_(0-182)_, AUC_(91-182)_, AUC_(0-210)_, apparent terminal half-life (following second injection), and the accumulation ratio based on a dosing interval of 91 days. All PK and statistical analyses were performed using SAS/STAT software. Comparative assessment of secondary PK measures focused on AUC_0-182_, trough concentrations, and C_max_ between the test and reference groups, but no formal hypothesis tests were prespecified for these comparisons.

Progesterone and E_2_ were measured locally at the DR and Oregon investigational sites using a Roche Cobas e411, a chemiluminescence-based automatic clinical platform (Roche Diagnostics). Measurement of MPA was conducted in a centralized fashion by PPD Development. Serum samples were prepared and immediately frozen at approximately −20°C at the investigational sites and then sent on dry ice to PPD Development where MPA levels were analyzed via a proprietary validated method using sensitive and selective high-performance liquid chromatography coupled with mass spectrometry. This method was applicable to the quantitation of MPA within a nominal range of 0.02 to 5.00 ng/mL.

## Results

A total of 52 healthy women were screened, and 42 of them (80.8%) were enrolled in the study: 36 (69.2%) participants were randomized into one of the three study groups, and six participants were purposefully enrolled into the 150 mg group at the DR site to achieve the target sample size of 24 in that group. The enrollment in the 300 mg and references groups was curtailed from 12 to nine women due to slower than expected enrollment at one of the sites.

**Figure 1:**
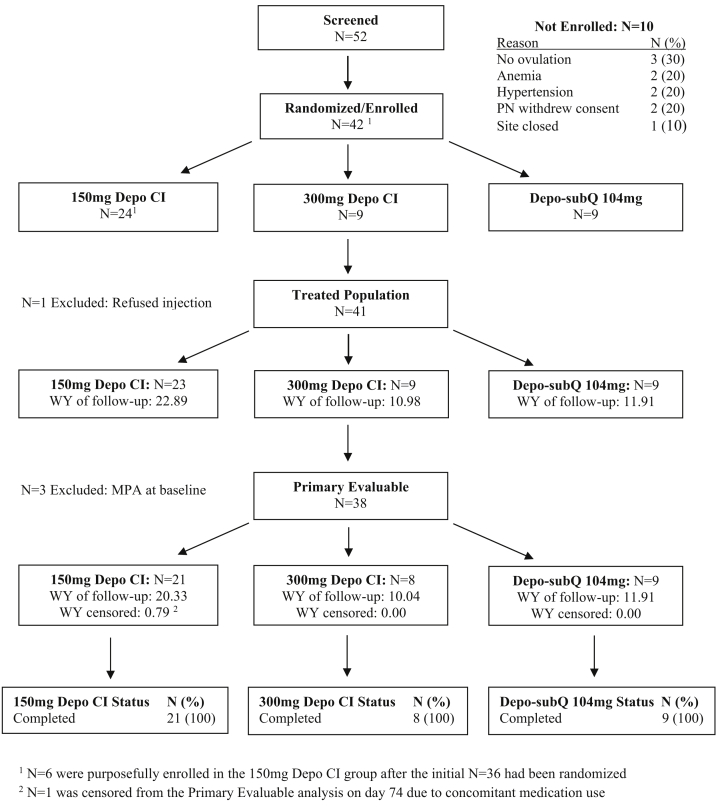
Participant disposition.

One participant in the 150 mg group refused injection after being randomized, and an additional three women had baseline MPA concentration exceeding 5% of their individual C_max_, leaving a total of 38 women evaluable for the primary PK and PD analyses (Fig. 1). One woman in the 150 mg group also had her data excluded after day 74 due to the use of a prohibited medication.

The median age of 38 evaluable participants was 34, ranging from 25 to 39 years; most (94.7%) were sexually active with a male partner; and most (92.1%) considered themselves biracial (Table 1). All but one participant (97.4%) had previously been pregnant. The most common contraceptive method used in the past was combined oral contraceptives (86.8%), followed by Depo-Provera (76.3%), condoms (68.4%), and implants (23.7%). Nearly all woman (94.7%) were sterilized. Median weight and BMI were 67.1 kg (47–83) and 26.5 kg/m^2^ (18–33), respectively, but were somewhat higher in the 300 mg group (76.6 kg and 30.6 kg/m^2^). There were no other meaningful differences between the treatment groups in the demographic and baseline characteristics.

**Table 1:** Summary of demographic characteristics.

Variable	150 mg, n = 21	300 mg, n = 8	Depo-SubQ 104, n = 9	Total, n = 38^a^
Age at baseline (y)				
Mean (SD)	32.8 (3.4)	33.5 (4.7)	35.3 (3.0)	33.6 (3.7)
Median (range)	33 (25–38)	34 (28–39)	36 (28–38)	34 (25–39)
Race, n (%)				
White	1 (4.8)	1 (12.5)	1 (11.1)	3 (7.9)
Biracial	20 (95.2)	7 (87.5)	8 (88.9)	35 (92.1)
Sexual activity status				
Active with male partner	19 (90.5)	8 (100)	9 (100)	36 (94.7)
Active with female partner	0 (0.0)	0 (0.0)	0 (0.0)	0 (0.0)
Abstinent	2 (9.5)	0 (0.0)	0 (0.0)	2 (5.3)
Weight (kg)				
Mean (SD)	65.8 (9.55)	75.5 (5.58)	61.9 (6.63)	66.9 (9.34)
Median (range)	66.3 (48 to 81)	76.6 (67 to 83)	63.3 (47 to 70)	67.1 (47 to 83)
Body mass index (kg/m^2^), n (%)				
<25	8 (38.1)	1 (12.5)	2 (22.2)	11 (28.9)
25–29	8 (38.1)	2 (25.0)	7 (77.8)	17 (44.7)
≥30	5 (23.8)	5 (62.5)	0 (0.0)	10 (26.3)
Mean (SD)	26.3 (3.99)	29.2 (3.70)	25.4 (2.87)	26.7 (3.85)
Median (range)	27.3 (18–32)	30.6 (24–33)	25.7 (19–29)	26.5 (18–33)
Ever pregnant, n (%)				
No	0 (0.0)	1 (12.5)	0 (0.0)	1 (2.6)
Yes	21 (100)	7 (87.5)	9 (100)	37 (97.4)
Contraceptives used in the past^b^: n (%)				
COCs	17 (81.0)	8 (100)	8 (88.9)	33 (86.8)
Implant	3 (14.3)	3 (37.5)	3 (33.3)	9 (23.7)
DMPA	14 (66.7)	7 (87.5)	8 (88.9)	29 (76.3)
Condoms	14 (66.7)	8 (100)	4 (44.4)	26 (68.4)
Contraceptive used in study: n (%)				
Condoms	0 (0.0)	0 (0.0)	1 (11.1)	1 (2.6)
Male sterilization	1 (4.8)	0 (0.0)	0 (0.0)	1 (2.6)
Female sterilization	20 (95.2)	8 (100)	8 (88.9)	36 (94.7)

*Note:* COC = combined oral contraceptives; DMPA = Depo-Provera; SD = standard deviation.

a Excludes data from 2 and 1 participant in the 150 mg and 300 mg groups, respectively, due to elevated MPA levels at baseline.

b Only methods reported by at least 20% of participants. Multiple responses may apply.

### Return to Ovulation

**Figure 2:**
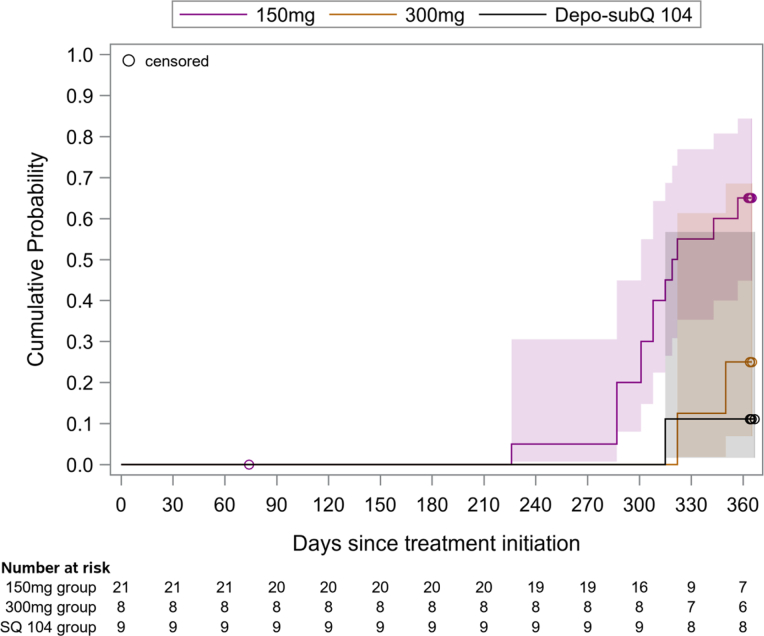
Kaplan-Meier estimates of cumulative probabilities of return to ovulation, with 95% confidence intervals and numbers at risk below the *x*-axis. The estimated probabilities at month 12 were 65%, 25%, and 11% in the 150 mg, 300 mg, and Depo-SubQ 104 groups, respectively (*P* = .009 for test of difference in distributions across groups). The earliest ovulation occurred on day 226 in the 150 mg group (median = 320 days). All doses were administered subcutaneously. The Depo-SubQ 104 group received a second injection on day 91.

None of the 38 evaluable women ovulated before month 7.5, with the earliest ovulation occurring in the 150 mg group on day 226 (7.5 months). The estimated probabilities of return to ovulation at month 12 were 65%, 25%, and 11% in the 150 mg, 300 mg, and Depo-SubQ 104 groups, respectively (Fig. 2). The woman in the 150 mg group who was censored from analysis on day 74 due to the use of a prohibited medication ovulated after month 12. All three participants who were excluded from the primary analysis due to detectable MPA at baseline ovulated between months 7.5 and 12.

### Supportive PD Analysis

Among participants who ovulated on and before month 12, the average maximum ovulatory P concentration of 10.3 ng/mL (range, 4.8–21.4) was lower than the average maximum P concentration of 14.5 ng/mL (range, 8.6–24.5) recorded among those participants at pretreatment.

Only one out of 21 evaluable women in the 150 mg group ovulated in the period when follicle growth was monitored by TVS. This participant had a rupture of the lead follicle (measured at 25 mm before rupture) on day 223 followed by elevated P of 7.4 ng/mL on day 226. Her day 224 MPA concentration was 0.186 ng/mL. Of the remaining 20 women, all but one had follicular activity documented by both elevated E_2_ levels and the growth of at least one lead follicle ≥ 15 mm (mean of maximum follicle size: 25.8 mm), but follicular rupture was not observed among any of these women during the 22- to 32-week assessment period.

### Pharmacokinetics

**Figure 3:**
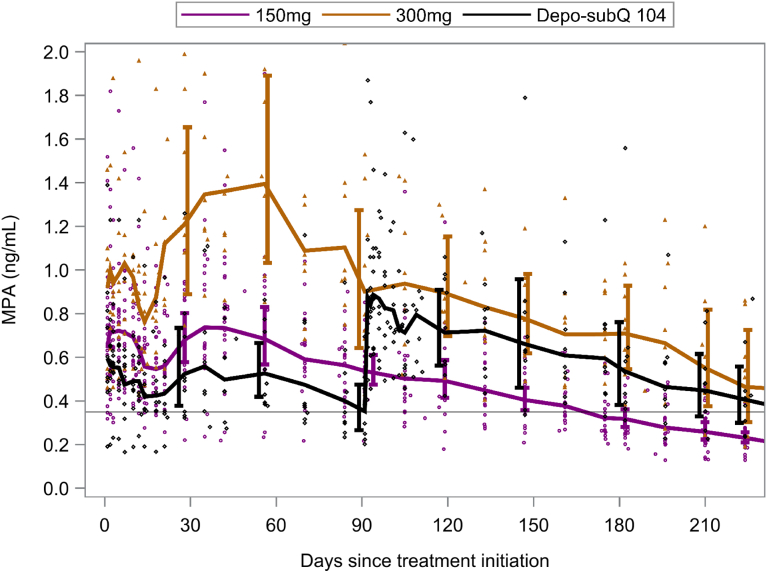
Geometric mean medroxyprogesterone acetate concentrations with 95% confidence intervals (shifted slightly for visibility). Reference line at 0.35 ng/mL is geometric mean at month 3 in the Depo-SubQ 104 group. All doses were administered subcutaneously. The Depo-SubQ 104 group received a second injection on day 91.

Geometric mean (GM) MPA concentrations through 7.5 months of treatment are presented in Figure 3. All but one participant (in the Depo-SubQ 104 group) achieved an MPA concentration above 0.2 ng/mL within 24 hours after injection. Only one woman in the 150 mg group had an observed MPA concentration below 0.2 ng/mL at 6 months after treatment initiation (0.181 ng/mL). However, her MPA concentration was 0.253 ng/mL at 6.5 months, and ovulation occurred at 9.4 months after treatment initiation. The GM C_max_ of 1.12 ng/mL in the 150 mg group was significantly greater than the GM C_max_ of 0.84 ng/mL in the first injection cycle of the reference group (GM ratio [GMR] = 1.33; 95% confidence interval [CI], 1.03–1.71), but was similar to the GM C_max_ of 1.07 ng/mL after the second injection of the reference group (GMR = 1.04; 95% CI, 0.82–1.32; Supplemental Table 1). The GM C_182_ of 0.32 ng/mL in the 150 mg group was 10% lower than the GM 3-month trough (C_91_) of 0.35 ng/mL for the reference group (GMR = 0.90; 95% CI, 0.67–1.21). The GM AUC_(0-182)_ of 98.2 ng∗days/mL in the 150 mg group was also approximately 10% lower than the GM of AUC_(0-182)_ 109.9 ng∗days/mL over two injection cycles of the reference group (GMR = 0.89; 95% CI, 0.71–1.13). In contrast, the GM C_max_ and AUC_(0-182)_ were significantly higher in the 300 mg group than either the 150 mg or reference groups. The GM C_182_ was also higher in the 300 mg group than in the reference group, but the difference was not statistically significant (GMR = 1.32; 95% CI, 0.86–2.03).

Although individual PK profiles were highly variable, each treatment exhibited biphasic absorption on average, with a lag of about 10 days for the second phase (Fig. 3). Among participants who ovulated, the highest observed MPA level at the time of ovulation was 0.186 ng/mL. Neither age nor BMI significantly impacted estimated PK parameters (data not shown).

### Safety and Acceptability

Injection site reactions were infrequent across all treatment groups, with a total of four participants reporting minor injection site reactions (e.g., redness, tenderness at the place of injection) that all resolved within 3 days after occurrence. The mean serum cortisol concentrations at day 7, month 3, and month 7.5 (150 mg group, 9.5–11.1 μg/dL; 300 mg group, 11.8–12.4 μg/dL; and reference group, 9.7–12.9 μg/dL) were indicative of adequate adrenal function and were generally comparable among the groups, with no meaningful changes from baseline or trends over time (data not shown).

The average (arithmetic mean) maximum E_2_ concentration observed at each 30-day interval in the 150 mg group remained between 100 and 150 pg/mL for about 7 months after injection, gradually increasing thereafter to 600 pg/mL at month 12 (Supplemental Fig. 2). Conversely, the average maximum E_2_ concentrations in the 300 mg and reference groups remained below 100 pg/mL for about 7 months after injection, increasing only to 300 pg/mL at month 12. The estimated probability of delayed return to ovulation (more than 12 months after injection) was 35.0% and 75.0% in the 150 and 300 mg group, respectively. In the reference group, the estimated probability was 88.9% (time from first injection) and 55.6% (time from second injection).

Comparable proportions of women in the 150 mg and reference groups reported spotting or irregular bleeding at month 3 (69.6% and 77.7%, respectively) and month 7.5 (73.9% and 88.9%, respectively). Amenorrhea was more common in the 300 mg group, with almost half of participants (44%) in this group reporting no bleeding at month 7.5 compared with 1% and 0% in the 150 mg and reference groups, respectively. Importantly, most (>75%) women in the test groups found their bleeding pattern acceptable. Given a choice between reinjections at every 1, 3, 6, or 12 months, most participants in both test groups would choose to receive injections every 6 months (>75%) and would use their assigned method if it proved to be effective for 6 months (>88%).

## Discussion

A single dose of 150 mg of Depo-Provera injected SC in the abdomen effectively suppressed ovulation for at least 7 months among all 21 evaluable women in this study, providing proof of concept that it may be an effective contraceptive method when injected every 6 months while reducing the overall exposure by approximately 30% compared to 6 months of Depo-SubQ 104 use. These compelling PD data were further supported by the PK results. All participants who received a single SC injection of 150 mg of Depo-Provera achieved MPA concentrations above 0.2 ng/mL within 24 hours after the injection, and all participants but one maintained MPA concentrations above that level for at least 6 months. The estimated 6-month MPA trough concentration for a 150 mg dose of Depo-Provera was 10% lower than the 3-month trough of the reference drug. However, this may not be clinically relevant given the lack of any pregnancies among 2,042 women using Depo-SubQ 104 for up to 1 year in three pivotal trials (9), which indicate that the 104 mg dose may itself be higher than necessary for a 3-month product.

The absence of ovulations in the first 7 months for a 150 mg dose of Depo-Provera suggests that there could be up to a 4-week window for reinjection, without relying on secondary mechanisms of action. Although the label for the reference drug provides for only a 1-week reinjection window, a longer interval would be a valuable programmatic attribute for an injectable contraceptive (14).

The supportive PD data showed that return of ovulation was characterized by lower P peak levels compared with baseline. Possible abnormal ovulation leading to luteal phase deficiency may, like other secondary mechanisms of action of MPA, contribute to a lower fertility after the SC administration of Depo-Provera, even after ovulation returns. This would be consistent with the data for Depo-SubQ 104 that suggest a gap between return to ovulation and return to fertility (9) and lends further support for extending the reinjection window up to 4 weeks.

While the GM maximum MPA concentration in the 150 mg group (1.12 ng/mL) was significantly higher than that of the reference group in the first injection cycle (0.84 ng/mL), it was comparable to GM maximum after the second injection (1.07 ng/mL) and substantially lower than historical peak MPA after intramuscular administration of Depo-Provera (3.73 ng/mL) (11). This, coupled with similar AUC _0-182_, alleviates safety concerns related to high MPA blood levels after SC administration of a 44% higher dose than Depo-SubQ 104.

With respect to follicular activity, circulating levels of E_2_ in the 150 mg group were on average consistent with midfollicular levels during the first 7 months postinjection. In contrast, the levels of E_2_ in the 300 mg and reference groups were lower and consistent with early to mid-follicular E_2_ levels. Less profound suppression of follicular activity and higher circulating levels of E_2_ in the 150 mg group may contribute to a better safety profile, reducing adverse effects associated with hypoestrogenism that are common among users of the approved Depo-Provera injectable products, specifically reduction in bone density. A more rapid relative return to ovulation is another potential benefit. In our study, only one of nine (11%) women who received two injections of Depo-SubQ 104 over 6 months had ovulated by 12 months after treatment initiation, compared with an estimated 65% in the 150 mg group at the corresponding time point. The rest of the safety data indicate that SC administration of Depo-Provera was safe and well tolerated; menstrual irregularities were common but in general acceptable.

Our trial has limitations. Specifically, most of the study participants were of African descent, and their BMI did not exceed 35 kg/m^2^ (class 1 obesity). We also exclusively used the abdomen as the site of injection, and the rate of absorption may differ for other injection sites (15). In addition, caution is required when interpreting these data due to the modest sample size, which was not designed to make definitive conclusions regarding efficacy or comparative PK. Whether our results are generalizable to a broader population and for other sites of injection remains to be established. Finally, the unblinded nature of the study introduced the possibility of bias. But the objective nature of the main study outcomes and the fact that the laboratory personnel conducting these assessments were blinded mitigated the potential impact of bias on validity of the study results.

Recent research has provided evidence that many women would prefer injectables that last longer (5). In our study, if given a choice between reinjections at every 1, 3, 6, or 12 months, most women preferred to receive injections every 6 months. These data validate previous findings of acceptability of and potential demand for an injectable with fewer reinjections.

A 6-month injectable contraceptive option would be a valuable addition to the contraceptive method mix, especially for underserved populations with restricted access to family planning. Our study suggests that such novel longer-acting injectable could be rapidly brought to market in a cost-efficient manner by repurposing Depo-Provera, thereby expediting the availability of a new contraceptive option.

### Conclusions

A 150 mg dose of Depo-Provera delivered SC in the abdomen effectively suppressed ovulation for at least 7 months among 21 women with BMI of 18–35 kg/m^2^ and exhibited a similar 6-month trough MPA concentration as the 3-month trough of Depo-SubQ 104. These data provide proof of concept that Depo-Provera (150 mg) may be a safe and effective contraceptive method when injected SC every 6 months, with up to a 4-week grace period for reinjections. The pregnancy prevention potential of this approach should be investigated further in a phase 3 efficacy trial.

## Supplementary data

Supplemental Figure 1 Schedule of follow-up procedures.

Supplemental Figure 2 Average maximum estradiol measurement in each 30-day period (±standard deviation shifted slightly for visibility). All doses were administered subcutaneously. The Depo-SubQ 104 group received a second injection on day 91.
