# Supplementary figures and images for: Clinical trial to evaluate pharmacokinetics and pharmacodynamics of medroxyprogesterone acetate after subcutaneous administration of Depo-Provera
Source: Fertil Steril. 2021 Apr;115(4):1035–43. doi: 10.1016/j.fertnstert.2020.11.002 (PMC8051852; doi:10.1016/j.fertnstert.2020.11.002)

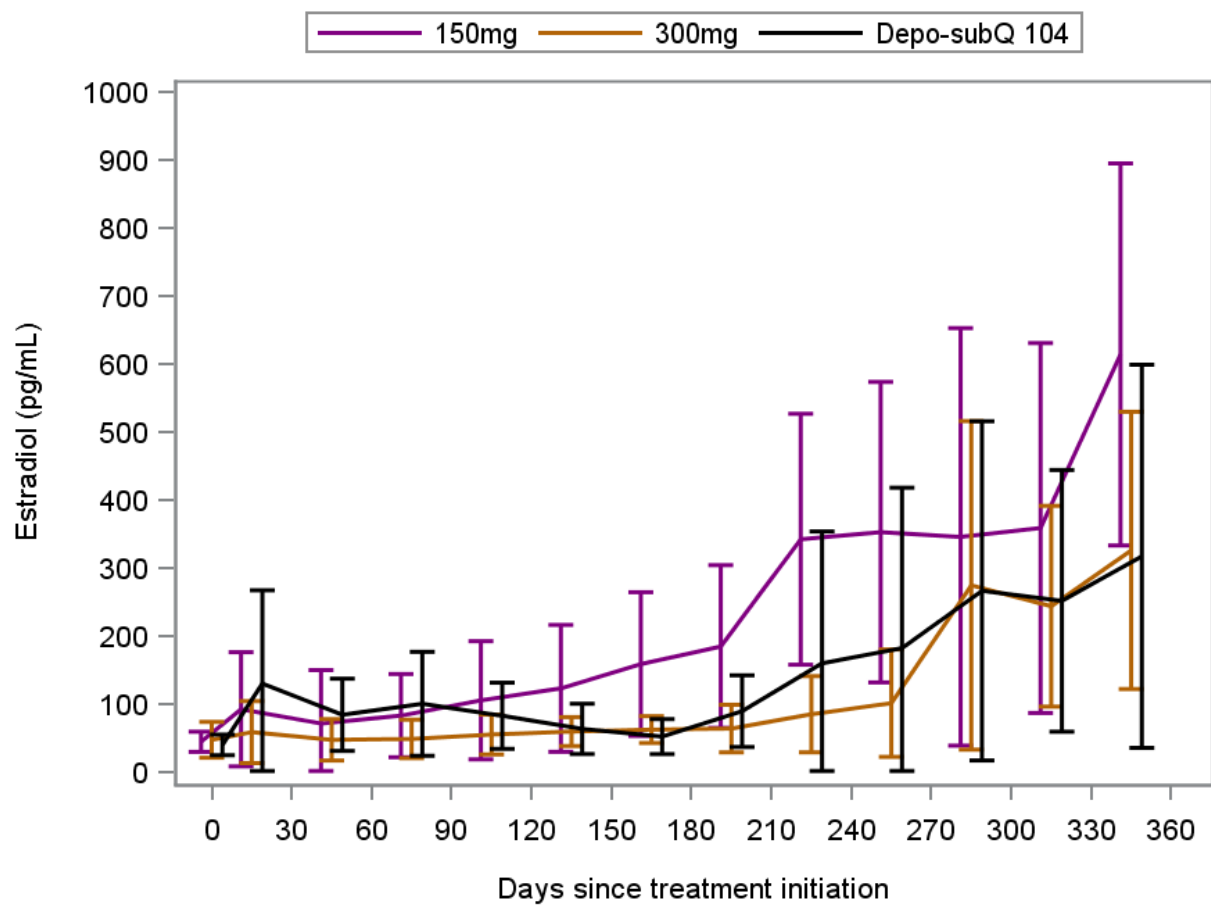

Supplement: Supplemental Figure 1 — Schedule of follow-up procedures. [file mmc1.pdf]
