# Supplementary material for: Clinical trial to evaluate pharmacokinetics and pharmacodynamics of medroxyprogesterone acetate after subcutaneous administration of Depo-Provera
Source: Fertil Steril. 2021 Apr;115(4):1035–43. doi: 10.1016/j.fertnstert.2020.11.002 (PMC8051852; doi:10.1016/j.fertnstert.2020.11.002)
Supplement: Supplemental Figure 2 — Average maximum estradiol measurement in each 30-day period (±standard deviation shifted slightly for visibility). All doses were administered subcutaneously. The Depo-SubQ 104 group received a second injection on day 91. [file mmc2.pdf]

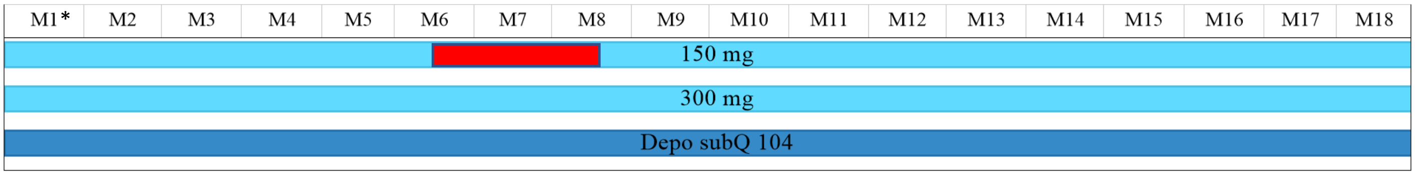

P/E2 on days 0, 7, 14, 28, 42; then at weeks 8, 10, 12; then weekly through week 32; then weekly until return of ovulation or through week 52, whichever was earlier. MPA on days 0, 1, 2, 3, 5, 7, 10, 12, 14, 18, 21; 28, 35, 42; then at weeks 8, 10, 12, 13, 15, 17, 19, 21, 23, 25, 26, 28, 30, 32; then every 28 days until return of ovulation, or week 52, whichever was earlier. If ovulation did not return before week 52, lab visits occurred at weeks 61-65 and 74-78 for additional P, E2 and MPA measurements.

P/E2 on days 0, 7, 14, 28, 42; then at weeks 8, 10, 12; 13 (re-injection), 15, 17, 19, 21, 23, 25, then weekly through week 32; then weekly until return of ovulation or through week 52, whichever was earlier. MPA on days 0, 1, 2, 3, 5, 7, 10, 12, 14, 18, 21, 28, 35, 42, 56, 70, 84, 91 (re-injection), 92, 93, 94, 96, 98, 101, 103, 105, 109; then at weeks 17, 19, 21, 23, 25, 26, 28, 30, 32; then every 28 days until return of ovulation or through week 52, whichever was earlier. If ovulation did not return before week 52, lab visits occurred at weeks 61-65 and 74-78 for additional P, E2 and MPA measurements.

Additional PD measurements: TVS, P and E2 performed twice a week during weeks 22, 23, 24, 25, 26, 27, 28, 29, 30, 31, 32; cervical mucus assessments performed when follicle of ≥15 mm detected by TVS.

* Month 1
