# Supplementary material for: Clinical trial to evaluate pharmacokinetics and pharmacodynamics of medroxyprogesterone acetate after subcutaneous administration of Depo-Provera
Source: Fertil Steril. 2021 Apr;115(4):1035–43. doi: 10.1016/j.fertnstert.2020.11.002 (PMC8051852; doi:10.1016/j.fertnstert.2020.11.002)
Supplement: Supplemental Table 1 [file mmc3.docx]

Supplemental Table 1. Pharmacokinetic parameter estimates^a^

|  | 150 mg | | 300 mg | | Depo-SubQ 104 | | Geometric mean ratio (95% confidence interval) | | |
| --- | --- | --- | --- | --- | --- | --- | --- | --- | --- |
| PK parameter | n | Geometric mean | n | Geometric mean | n | Geometric mean | 300 vs. 150 mg | 150 vs. Depo-SubQ 104 | 300 mg vs. Depo-SubQ 104 |
| C_max_^b^ | 21 | 1.12 | 8 | 1.74 | 9 | 0.84 | 1.56 (1.22, 2.00) | 1.33 (1.03, 1.71) | 2.07 (1.49, 2.89) |
| C_max_^c^ |  |  |  |  | 9 | 1.07 |  | 1.04 (0.82, 1.32) | 1.62 (1.20, 2.18) |
| T_max_^b^ | 21 | 9.97 | 8 | 20.30 | 9 | 5.92 | 2.04 (0.63, 6.61) | 1.69 (0.49, 5.86) | 3.43 (0.59, 20.0) |
| C_91_ | 20 | 0.54 | 8 | 0.90 | 9 | 0.35 | 1.68 (1.25, 2.25) | 1.52 (1.15, 2.01) | 2.56 (1.66, 3.93) |
| C_182_ | 20 | 0.32 | 8 | 0.71 | 9 | 0.54 | 2.23 (1.68, 2.95) | 0.59 (0.44, 0.81) | 1.32 (0.86, 2.03) |
| C_182_ vs C_91_^d^ | 20 | 0.32 | 8 | 0.71 | 9 | 0.35 |  | 0.90 (0.67, 1.21) | 2.01 (1.36, 2.97) |
| C_210_ | 20 | 0.26 | 8 | 0.55 | 9 | 0.45 | 2.13 (1.48, 3.07) | 0.58 (0.41, 0.81) | 1.23 (0.77, 1.98) |
| AUC_(0-91)_ | 21 | 59.88 | 8 | 107.13 | 9 | 45.20 | 1.79 (1.40, 2.29) | 1.32 (1.03, 1.70) | 2.37 (1.70, 3.30) |
| AUC_(0-182)_ | 21 | 98.20 | 8 | 183.02 | 9 | 109.86 | 1.86 (1.47, 2.36) | 0.89 (0.71, 1.13) | 1.67 (1.25, 2.22) |
| AUC_(0-210)_ | 21 | 105.98 | 8 | 202.31 | 9 | 123.93 | 1.91 (1.51, 2.41) | 0.86 (0.68, 1.08) | 1.63 (1.23, 2.17) |
| AUC_(0-inf)_ | 21 | 140.79 | 8 | 266.46 | 9 | 209.06 | 1.89 (1.44, 2.48) | 0.67 (0.51, 0.90) | 1.27 (0.83, 1.96) |
| Half-life^c^ | 21 | 76.21 | 8 | 68.61 | 9 | 83.83 | 0.90 (0.54, 1.51) | 0.91 (0.50, 1.66) | 0.82 (0.37, 1.81) |

*Note:* Units: AUC = area under the curve (days*ng/mL); C= concentration (ng/mL); HL = XXX; T = time.

^a^Excludes 2 and 1 participant’s data from the 150 mg and 300 mg groups, respectively, due to elevated MPA levels at baseline.

^b^Based on first injection cycle for Depo-SubQ 104 group.

^c^Based on second injection cycle for Depo-SubQ 104 group.

^d^Comparing geometric mean concentration at day 182 in 150 mg and 300 mg groups to geometric mean concentration at day 91 in the Depo-SubQ 104 group.
